# Tau Protein Alterations Induced by Hypobaric Hypoxia Exposure

**DOI:** 10.3390/ijms25020889

**Published:** 2024-01-10

**Authors:** Eduardo Pena, Rocio San Martin-Salamanca, Samia El Alam, Karen Flores, Karem Arriaza

**Affiliations:** High Altitude Medicine Research Center (CEIMA), Arturo Prat University, Iquique 1110939, Chile; eduardopena@unap.cl (E.P.); rsanmartins@protonmail.ch (R.S.M.-S.); kfloresu@unap.cl (K.F.); karriaza@unap.cl (K.A.)

**Keywords:** tauopathies, hypoxia, high altitude, Alzheimer’s disease, neurology

## Abstract

Tauopathies are a group of neurodegenerative diseases whose central feature is dysfunction of the microtubule-associated protein tau (MAPT). Although the exact etiology of tauopathies is still unknown, it has been hypothesized that their onset may occur up to twenty years before the clear emergence of symptoms, which has led to questions about whether the prognosis of these diseases can be improved by, for instance, targeting the factors that influence tauopathy development. One such factor is hypoxia, which is strongly linked to Alzheimer’s disease because of its association with obstructive sleep apnea and has been reported to affect molecular pathways related to the dysfunction and aggregation of tau proteins and other biomarkers of neurological damage. In particular, hypobaric hypoxia exposure increases the activation of several kinases related to the hyperphosphorylation of tau in neuronal cells, such as ERK, GSK3β, and CDK5. In addition, hypoxia also increases the levels of inflammatory molecules (IL-β1, IL-6, and TNF-α), which are also associated with neurodegeneration. This review discusses the many remaining questions regarding the influence of hypoxia on tauopathies and the contribution of high-altitude exposure to the development of these diseases.

## 1. Introduction

Neurodegenerative diseases (NDDs) are among the top ten causes of death worldwide [1] and the top three causes of death in the Americas and Europe [2]. These diseases are characterized by the loss of neuronal function and the continuous deterioration of cognitive ability [3]. Tauopathies are neurodegenerative diseases characterized by abnormalities in the tau protein, which is responsible for the stabilization of microtubules and proper microtubule assembly in neurons in the central nervous system (CNS) [3,4,5]. Dysfunction of tau causes its uncoupling from microtubules and subsequent aggregation in oligomers, causing synaptic dysfunction, inflammation, and cell death [5,6]. Although the cause of these abnormalities is unknown, their risk is determined, in part, by environmental and genetic factors and is closely related to advanced age [3].

Various factors, such as genetic mutations, posttranslational modifications, and interactions with other proteins, trigger pathological changes in tau [5,7,8]. One of these factors is oxygen (O_2_) deficiency or hypoxia, which can be caused by several medical conditions, such as obstructive sleep apnea (OSA), vascular diseases, and exposure to high altitudes, and can contribute to the development of tauopathies [9,10,11,12,13]. Hypobaric hypoxia results from decreases in barometric pressure and the partial pressure of O_2_ at high altitudes (over 2500 m) and reduces the bioavailability of O_2_ in alveolar tissues and cells, thus altering aerobic processes [14]. Some of the principal pathologies associated with hypobaric hypoxia exposure are acute mountain sickness, high-altitude pulmonary edema, high-altitude cerebral edema [15], high-altitude pulmonary hypertension [16], and chronic mountain sickness [17].

A relationship has been found between the body’s response to tau aberrations and exposure to hypoxia because they activate similar molecular mechanisms and neuronal biomarkers, and this relationship has been studied mainly in models of hypoxia, such as OSA [18,19].

The most prevalent tauopathy is Alzheimer’s disease (AD), which currently affects approximately 40 million people worldwide [20]. There is an evident correlation between the gradual aging of the population and an increase in the incidence of this disease, which is why it represents an alarming public health concern. Therefore, it is imperative for the scientific community to find solutions and develop effective strategies for the early diagnosis and treatment of these diseases.

To our knowledge, the majority of studies on the direct relationship between tauopathies and hypoxia have focused on models of OSA-associated hypoxia, and there have been fewer studies on other models of hypoxia. In particular, hypobaric hypoxia has particular relevance in Chile because many people in the country live or work in the Andes mountains and are exposed to hypobaric hypoxia, that is, O_2_ deficiency resulting from high-altitude exposure. Therefore, the objective of this study was to identify tau alterations induced by hypobaric hypoxia exposure and their association with the development of tauopathies.

## 2. Tau Pathology Mechanisms

Neurodegenerative diseases are characterized by the gradual, progressive, and irreversible deterioration of psychomotor function, cognition, and memory caused by the loss of neuronal function and neuronal death. These disorders can be sporadic, that is, they can be acquired at various stages of life, or familial, with familial disease accounting for a smaller number of cases. Although neurodegeneration is more common in older individuals, it is not part of the normal aging process [21,22].

Tauopathies are NDDs that are characterized by tau protein alterations, neuroinflammation, and the intracellular accumulation of tau oligomers. Over time, tau oligomers become more voluminous, forming fibrillar aggregates known as paired helical filaments (PHFs) and neurofibrillary tangles (NFTs), which impede synaptic and neuronal functions [5]. The presence of these aggregates is the characteristic histopathological feature of tauopathies [3,6,23,24,25]. To date, the etiology of these diseases is unknown, but various studies have indicated that it involves multiple factors, such as genetic abnormalities, unhealthy habits, exposure to injurious stimuli, and accidents [13,26,27,28,29,30,31,32].

Tauopathies can be classified as primary or secondary tauopathies. While tau impairment is the key feature of primary tauopathies, secondary tauopathies are characterized by tau protein aggregation and tau oligomer formation [33]. The most prevalent tauopathies are secondary tauopathies, e.g., AD, Parkinson’s disease (PD), and Huntington’s disease (HD), which account for more than 80% of tauopathy cases worldwide [33,34]. There are several primary tauopathies, such as argyrophilic grain disease (AGD), Creutzfeld–Jakob disease (CJD), and corticobasal degeneration (CBD), in which tau alterations play a central role in neurodegeneration (Table 1); however, as the incidence of these diseases is lower than that of secondary tauopathies, this review will focus on secondary tauopathies.

AD is the most common age-related neurodegenerative disease and is characterized by synaptic and neuronal loss, progressive dementia, and cognitive impairment. The key pathological hallmark of AD is the accumulation of intracellular neurofibrillary tangles composed of pTau and extracellular β-amyloid plaques, and the hippocampus and cerebral cortex are the most widely affected structures in AD [23,28,44]. PD is a neurodegenerative disease characterized by motor-related symptoms such as resting tremor and balance and gait disturbances, but PD also causes nonmotor symptoms such as cognitive decline. In PD, misfolded α-synuclein protein and tau deposits are found in dopaminergic neurons. Although the specific cause of PD is not known, there are several environmental and genetic factors (for instance, dominant or recessive mutations in genes such as LRRK2 and parkin) that can cause PD pathogenesis [45,46]. HD is an autosomal dominant neurodegenerative disorder that usually appears in middle-aged individuals and is associated with symptoms such as involuntary movements, cognitive impairment, and neuropsychiatric disturbances. It is caused by an expanded CAG repeat in exon 1 of the huntingtin gene (*HTT*) on chromosome 4p16.3, resulting in an abnormal polyglutamine (polyQ) expansion of the amino-terminal region of the huntingtin protein. The mutated huntingtin protein (mHtt) misfolds and oligomerizes in all cells of the body. Recent investigations have suggested that this HD is a secondary tauopathy, as mHtt has been confirmed to participate in tau hyperphosphorylation via calcineurin [47].

At the cellular level, microtubules are key organelles involved in cytoarchitecture, substance transport, and the modulation of genetic processes. They are composed of tubulin heterodimers and stabilized by microtubule-associated proteins (MAPs) [48]. In CNS neurons, the predominant MAP is tau, or microtubule-associated protein tau (MAPT), which is involved mainly in microtubule assembly and is mostly concentrated in neuronal axons [6,8,22,49,50].

Phosphorylation is the most important posttranslational modification of tau, as it modulates microtubule stabilization [7,48,51]; tau is coupled and uncoupled from microtubules through dephosphorylation and phosphorylation by phosphatases and kinases, respectively, and under physiological conditions, there is a dynamic balance between microtubule-bound tau and free tau [51,52].

Serine/threonine phosphatases (protein phosphatases, PPs) remove phosphate groups from proteins. Among the four PPs (PP1, PP2A, PP2B, and PP2C), PP2A is considered the most important due to its key role in the control of microtubular dynamics and the regulation of tau phosphorylation [53,54,55]. Furthermore, studies have shown that the inhibition of PP2A renders tau prone to hyperphosphorylation by kinases [55].

**Figure 1:**
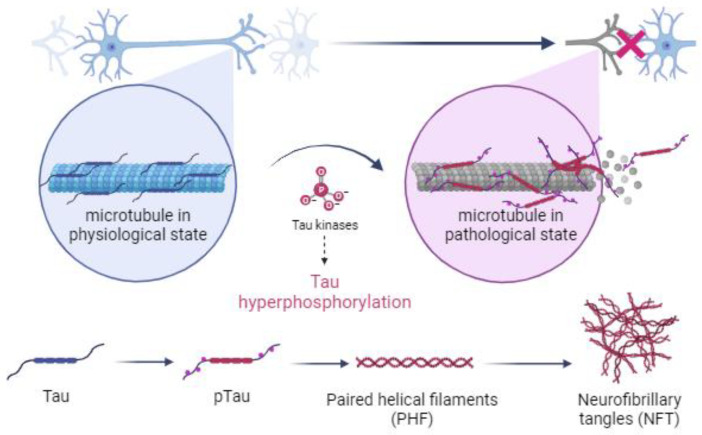
Illustration of the mechanism by which kinases cause hyperphosphorylation of tau and the subsequent formation of paired helical filaments (PHFs) and neurofibrillary tangles (NFTs) in neuronal cells of the central nervous system during the development of tauopathies. Created with BioRender.com.

Under pathological conditions, specifically during the development of tauopathies, genetic mutations or persistent environmental stimuli activate metabolic cascades that lead to changes in the cellular environment, which inhibit the activity of phosphatases and promote the overexpression of kinases, disrupting the balance between the two forms of tau by causing the hyperphosphorylation of tau and inducing a conformational change in tau that prevents its binding to microtubules [5,24,55,56,57]. Hyperphosphorylated tau is a distinctive feature of tauopathies (Figure 1) [30,58].

**Figure 2:**
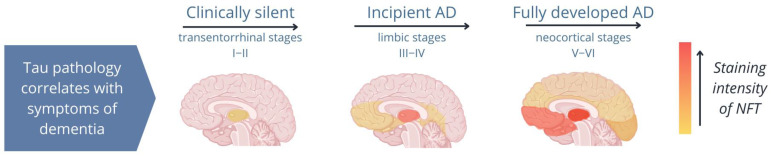
Image of the distribution of neurofibrillary tangles (NFTs) during different stages of Alzheimer’s disease (AD). In AD, tau pathology begins in the hippocampus and spreads from the limbic system to the neocortex. A patient’s symptoms depend on the areas of the brain to which tau aggregates (NFTs; shown in orange) spread [61].

Several studies have shown that once pTau is formed, it is unable to bind to microtubules but instead binds to other pTau and tau molecules, forming fibrillar aggregates, which cause structural and functional abnormalities in neurons and impair synaptic function [7,22,28,59,60]. Importantly, pTau aggregations occur in both healthy individuals and diseased individuals; however, while fibrillar aggregates are present in the hippocampus of healthy elderly individuals, they spread from the hippocampus to the neocortex in patients with tauopathies (Figure 2; Ariza et al. [61]). Therefore, the spread of tau pathology is a reliable indicator of the disease stage in tauopathies [21,62,63,64,65].

However, there are some controversies regarding pTau and fibrillar aggregates; some studies suggest that pTau is more harmful to neurons than fibrillar aggregates, as it is thought that the formation of aggregates could be an adaptive mechanism for scavenging pTau, slowing its spread, and minimalizing its effect on tau [8,24,66]. This phenomenon has been validated by research on cell lines and animals [67,68]. However, there is a general consensus that fibrillar aggregates have a negative effect. Considering this controversy, additional research is needed to confirm the effect of pTau.

### 2.1. Kinase-Mediated Dysregulation

**Figure 3:**
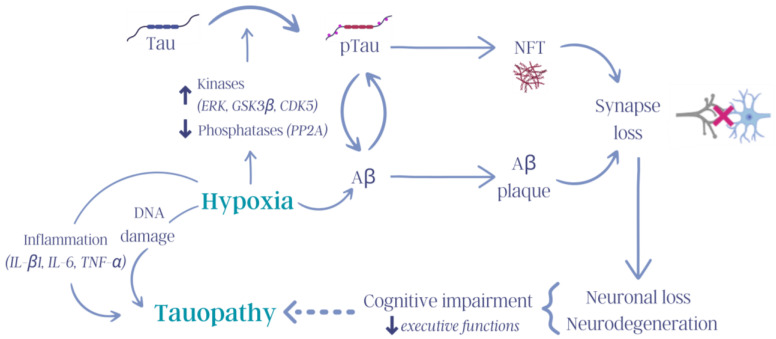
Influence of hypoxia on tauopathies. Hypoxia activates numerous molecular mechanisms in the body that trigger, for example, increased inflammation, increased DNA damage, kinase and phosphatase dysregulation, and tau pathology, thus contributing to the cognitive deterioration observed in tauopathies (tau: tau protein; pTau: hyperphosphorylated tau protein; ERK: extracellular signal-regulated kinase, GSK3β: glycogen synthase kinase 3 β; CDK5: cyclin-dependent kinase 5; PP2A: protein phosphatase 2 A; NFTs: neurofibrillary tangles; Aβ: β-amyloid protein; IL-β1: interleukin-β1; IL-6: interleukin-6; TNF-α: tumor necrosis factor-α). Created with Canva (Sydney, Australia).

The kinases that regulate tau phosphorylation are classified into three different groups: proline-directed kinases (PDPKs), non-proline-directed protein kinases (non-PDPKs), and tyrosine protein kinases (TPKs) [69] (Figure 3).

PDPKs phosphorylate tau at Ser/Thr residues under both physiological and pathological conditions [69]. The two most important kinases in this group are cyclin-dependent kinase 5 (CDK5) and glycogen synthase kinase 3 β (GSK3β), which play important roles in the hyperphosphorylation of tau, as indicated by the fact that their inhibition decreases the formation of fibrillar aggregates [70,71]. Notably, there is positive feedback between CDK5 and GSK3β, as CDK5 increases the activity of GSK3β [72]. Interestingly, several studies have shown that altered CDK5 activity elicits changes similar to those observed in AD [56,73,74,75,76,77,78]. Furthermore, it has been demonstrated that MAPKs (JNK 1/2/3, p38, and ERK 1/2) phosphorylate almost all of the residues of tau, whose phosphorylation is associated with AD [69,79].

Non-PDPKs, which also phosphorylate Ser/Thr residues, phosphorylate tau at key sites related to AD pathology [52,70,80,81,82]. In tauopathies, calcium homeostasis is dysregulated [83]; thus, researchers have shown interest in studying the calcium-dependent kinase Ca^2+^/calmodulin-dependent protein kinase II (CaMKII), which phosphorylates tau at residues located in microtubule-binding domains [84,85], resulting in the formation of fibrillar tau aggregates (PHFs and NFTs) in the hippocampus [62,86,87,88].

TPKs, which include c-Abelson (c-Abl), phosphorylate tyrosine residues. SFKs, which participate in the development of tauopathies to a lesser degree than other more studied tau kinases [89], phosphorylate tau at Tyr 18, 29, 197, 310, and 394, which are related to tauopathies, and at residues that are phosphorylated under physiological conditions, such as Tyr 394 [89].

### 2.2. Interaction between Tau and Other Proteins

Several proteins can affect the function of tau by altering it in different regions, competing with other proteins to bind tau, and preventing its normal binding activity and function [5]. It is important to consider the roles of other proteins in the pathogenesis of tauopathies; some of these proteins are mentioned below.

β-amyloid (Aβ) is one of the most important proteins in AD, as misfolded Aβ forms aggregates called β-amyloid plaques that accumulate extracellularly. The aberrant folding of Aβ is due to erroneous cleavage of the protein, which generates the peptides Aβ40 and Aβ42, accounting for ~80–90% and ~5–10%, respectively, of all Aβ [90]. Aβ42 is more hydrophobic and fibrillogenic and has a greater propensity for aggregation; therefore, it is the most harmful species of Aβ [20]. There is a form of feedback between Aβ and tau; therefore, deregulation of either protein is very harmful. Aβ plays a fundamental role in the phosphorylation of tau, both in vitro and in vivo [73,75,91], and negatively influences the metabolic pathway of CDK5.

There is evidence that tau and Aβ interact during the progression of AD, with Aβ accelerating tau alterations and the formation of NFTs [92] and tau playing a role in the toxic effects of Aβ [93,94]. Emerging evidence on this topic suggests that both proteins have a common or positive feedback mechanism. This suggests that both proteins are important for monitoring NDD progression.

α-Synuclein, a postsynaptic protein abundantly expressed in neurons that has also been associated with tauopathies, is specifically relevant in Parkinson’s disease. The accumulation of hyperphosphorylated tau and NFT formation affect axonal transport, and pTau has been widely observed to be localized in Lewy bodies, abnormal α-synuclein deposits characteristic of PD. This positive correlation between α-synuclein and tau levels has led to the hypothesis that there is an interaction between these two proteins during the course of the disease [5,31,95].

Specifically, in Huntington’s disease (HD), a correlation has been found between the levels of tau and huntingtin (Htt), a ubiquitous protein abundantly expressed in the brain and testes. While the physiological function of Htt is still unclear, it is presumed that it is a pleiotropic protein that participates in cytoarchitecture, among other processes [96]. Mutant Htt (mHtt) and the missplicing of Htt lead to pathogenic abnormalities in tau, affecting the proportions of its isoforms and total content, promoting the hyperphosphorylation of tau, and causing GSK3β alterations [47,71,97,98].

## 3. Hypoxia and Dysregulation of Tau

Hypoxia refers to a deficit in O_2_ supply caused by any kind of alteration, either environmental or organismal, that disturbs the uptake, transport, and bioavailability of O_2_ in the cell [99]. This condition induces pathophysiological abnormalities that particularly damage the CNS, impairing cognition and memory [10,100]; therefore, it represents one of the most important risk factors for the development of diseases such as AD, PD, and HD [10,101]. However, as mentioned above, studies indicate that the development of neurodegenerative diseases may have multifactorial causes, with several factors interacting with each other, such as hypoxia, certain cardiovascular conditions, abnormal accumulation of proteins in the brain, blood–brain barrier alterations, and aging [12,102].

Hypoxia exacerbates tau pathology, promoting the formation of protein aggregates (such as Aβ plaques and NFTs) and causing cellular death, oxidative stress, and synapse loss [103,104,105]. Both oxidative stress and tau hyperphosphorylation play key roles in the pathophysiology of tauopathies [106,107]. Importantly, longer exposure to hypoxia results in more severe cellular damage [10,77,103,104,108]. Although the entire brain is affected by hypoxia, the most affected structure is the hippocampus because blood flow in this region is lower than that in other areas of the brain [109] and because fibrillar aggregates form first in this brain region.

Additionally, intermittent hypoxia exposure seriously affects the brain. It is linked to the hyperphosphorylation of tau at Ser and Thr residues and, thus, increases the activation of GSK3β and inactivates PP2A in the hippocampus [57]; these changes are directly related to neurodegeneration, cognitive impairment, and the development of tauopathies. The different types of hypoxia and their relationships with neurodegenerative diseases are detailed below, with a special emphasis on hypobaric hypoxia.

## 4. Hypobaric Hypoxia

As geographical altitude increases, especially above 2500 m above sea level, changes in atmospheric conditions occur, such as a reduction in barometric pressure, which generates a decrease in the partial pressure of gases. This phenomenon, which is called hypobaric hypoxia, has a pathophysiological effect because a decrease in arterial O_2_ pressure impedes O_2_ uptake and decreases O_2_ bioavailability in the lungs, as there is not enough alveolar pressure to diffuse the required amount of O_2_; this leads to reduced O_2_ levels in the blood, tissues, cells, and mitochondria [110].

Many people worldwide are exposed to high altitude, whether through participation in work or recreational activities or as permanent residents in high-altitude regions. Currently, 129.91 million people live more than 2500 m above sea level worldwide, and in Chile, approximately 78,000 people live above this altitude [111], not including those who travel to the country for work or tourism.

Hypobaric hypoxia has both short-term and long-term impacts and can have pathological effects; however, the severity of the neuronal damage induced by hypobaric hypoxia increases with exposure time [112]. The effects of hypobaric hypoxia include neurophysiological abnormalities such as poor sleep quality, dizziness, nausea, ataxia, and cognitive impairment [113,114]. Hypobaric hypoxia leads to neuroinflammation, oxidative stress, loss of muscle mass, and increased cytokine expression [65,115], ultimately leading to neurodegeneration in different brain regions. In particular, hypobaric hypoxia causes cellular damage in the hippocampus comparable to that observed in elderly individuals [64,112]. In addition, a study in transgenic mice revealed that exposure to hypobaric hypoxia during the gestation period is related to an increased risk of neurodegenerative diseases such as AD [116].

### 4.1. Acute Hypobaric Hypoxia (AHH)

In acute hypobaric hypoxia, the body is exposed to high altitudes for a short period, i.e., for a few hours or days, with a subsequent return to normobaric conditions. Studies in humans have shown that even short episodes of hypobaric hypoxia exposure (3450 m, 24 h) impair executive functions (memorization, organization, and language, among others) and cause memory deficits that are reversible when individuals return to normoxic conditions [117]. These findings are consistent with the results of studies conducted in Sprague–Dawley rats; that is, acute hypobaric hypoxia (6000 m, 3 days) causes the hyperphosphorylation of tau at Ser396, Ser262, Thr231, and Thr181 in the hippocampus, which has been related to impaired performance on memory tasks [118]. Regarding the time of high-altitude exposure required to cause acute hypobaric hypoxia, a recent study of male mice (C57BL/6J) exposed to an altitude of 7000 m for 1, 3, or 7 days revealed impaired learning and memory, with significant changes occurring on days 1 and 3. In addition, 739, 452, and 183 differentially expressed genes (DEGs) were identified in the day 1 group, day 3 group, and day 7 group, respectively, compared to the control group. Importantly, in all groups, neurological impairment was associated with oxidative stress, inflammatory responses, and synaptic plasticity, and neurological deficits were attenuated in the day 7 group. This study suggests that hypobaric hypoxia initially causes neurological impairment, followed by gradual acclimatization over time [119].

Acute hypoxia can also occur under normobaric conditions, and research on this type of acute hypoxia has contributed to the understanding of the effect of hypobaric hypoxia exposure. An in vivo study (C57 transgenic mice; hypoxia: 8% O_2_; 0, 2 and 4 h) and in vitro studies in cell lines (E17 cells, 1% O_2_; 0, 2 and 4 h; SH-SY5Y cells, 8% O_2_, 12 h) showed that acute hypoxia alters phosphorylation through the activation of ERK and that neither CDK5 nor GSK3 seem to play significant roles in these effects. It is hypothesized that ERK responds more quickly to hypoxic stimuli than CDK5 and GSK3, the main kinases involved in the abnormal phosphorylation of tau [120]. These results were also confirmed by Zhang et al. [121], who used similar parameters (7% O_2_, 24 h) to study the effect of hypoxia in wild-type and transgenic mouse strains (APP^swe^/PS1^dE9^), in which tau is hyperphosphorylated at Thr181 and Thr213 and Aβ is upregulated. However, studies in humans who practice freediving have shown that acute hypoxia increases the total levels of tau (tTau) and Aβ in plasma, potentially implicating these proteins in the molecular pathogenesis of AD [122].

Together, these findings suggest that acute hypoxia causes alterations that promote tau and Aβ pathology related to tauopathies in animal and cell models as well as in humans.

### 4.2. Chronic Hypobaric Hypoxia (CHH)

CHH is a condition caused by hypobaric hypoxia exposure for months or years. In CHH, the first change in the brain is angiogenesis; specifically, a study in rats exposed to hypobaric hypoxia (5500 m) for more than 3 weeks showed an increase in the hematocrit level and microvessel density in the cerebral cortex, frontal motor cortex, frontal sensory cortex, parietal motor cortex, parietal sensory cortex, hippocampus, striatum, and cerebellum. This indicates that chronic hypobaric hypoxia elicits alterations in brain morphology aimed at increasing O_2_ bioavailability in the brain [123]; this finding was corroborated by a subsequent study by Boero et al. [124], which revealed that angiogenesis in the mouse brain is induced by chronic hypobaric hypoxia exposure.

CHH activates mechanisms related to inflammation, tau hyperphosphorylation, cognitive decline, damage to brain and cell structures, and biological aging. Studies on humans, rats, and mice have yielded similar results regarding the deleterious effects of CHH. For example, increases in the levels of cytokines, particularly interleukin-β1 (IL-β1), interleukin-6 (IL-6), and tumor necrosis factor-α (TNF-α), which induce neuroinflammation and alter glial cell (astrocytes, oligodendrocytes, and microglia) function and are related to neurodegeneration in the hippocampus (Figure 3), have been observed after CHH exposure [65,112].

As mentioned before, CHH accelerates biological aging by altering the expression of markers related to aging (SNAP25, Tau, Sod2, APOE, and S100A9) [112], promoting tau hyperphosphorylation [125], and causing cognitive impairment [112,117,125].

These findings indicate that there is a relationship between the neuronal damage induced by hypobaric hypoxia, the hyperphosphorylation of tau, and cognitive deterioration, and that the detrimental effects of hypobaric hypoxia increase in severity as the duration of hypoxia exposure increases [117].

Studies on neurological impairment in people living at high altitudes are lacking. However, a recent study by Stacey and colleagues showed that the cognitive function of native highlanders from Cerro de Pasco (4300 m) was not altered compared with that of a lowlander group, whereas cognitive impairment was observed in lowlanders after two weeks of high-altitude exposure (4300 m) [126]. These findings are consistent with a previous study in rats exposed to hypobaric hypoxia (6100 m) for 3, 7, 14, and 21 days, which showed that memory impairment was alleviated and antioxidant levels were decreased on day 21 compared to day 7 [127]. However, additional studies are necessary to confirm these findings.

### 4.3. Chronic Intermittent Hypobaric Hypoxia (CIHH)

Another form of hypobaric hypoxia involves intermittent exposure to hypoxic stimuli, specifically days of exposure to hypobaric hypoxia followed by days under normoxic conditions, over a long period of time. This type of hypobaric hypoxia condition has been termed chronic intermittent hypobaric hypoxia (CIHH) [113]. Interestingly, compared with continuous hypoxia, intermittent exposure to hypoxia may have a more pronounced adverse effect on neuronal function and integrity [128]. For example, studies in Kunming mice exposed to CIHH (8 h/day, simulated altitude of 5500 m, for 28 days) showed significant impairment in memory and spatial learning and increased phosphorylation of tau at Thr181, Ser262, Ser202, Thr205, and Ser396 in the hippocampus and cerebral cortex [129]. Furthermore, exposure to CIHH during the gestation period was found to increase tau hyperphosphorylation (Ser396), affecting the synaptic ultrastructure and increasing the risk of AD, especially in genetically predisposed individuals [116]. CIHH was shown to increase pTau levels and, subsequently, cause glial alterations (Figure 3) [130].

To our knowledge, very little research has been performed on CIHH. However, it seems to be one of the most relevant types of hypoxia due to its pronounced deleterious effects.

There are controversies regarding the effect of hypoxia because, paradoxically, hypoxic pre- and post-conditioning can be used as a therapeutic strategy for certain diseases. For example, mild hypobaric hypoxia (three episodes of 360 Torr, 2 h, every 24 h) can exert a neuroprotective effect by reducing lipid oxidation and DNA fragmentation resulting from severe hypobaric hypoxia exposure (180 Torr, 3 h) in Wistar rats [131]. Acute intermittent hypoxia (ten daily episodes of 10.5% O_2_ for 5 min, with normoxic intervals of 5 min) has been shown to have a neuroprotective effect and promote neuroplasticity in Sprague–Dawley rats after daily exposure for 7 days, but to induce tau hyperphosphorylation in the hippocampus and cerebral cortex after daily exposure for 28 days [132]. In transgenic mice (C57Bl/6J), exposure to 10% O_2_ for 90 s (interval of 90 s, 20 hypoxic episodes per hour, 8 h/day for 14, 21, and 35 days; CHH) exerts a neuroprotective effect; however, if the O_2_ percentage is decreased to 6%, marked damage to neuronal tissue occurs [133]. Exposure to hypoxia combined with reoxygenation therapy can attenuate hyperphosphorylation of tau (Ser396, Ser262, Thr231, and Thr181) after exposure to hypobaric hypoxia, suggesting that it may be a therapeutic technique for individuals exposed to high altitudes [118].

On the other hand, a study in Kunming mice exposed to chronic intermittent high-altitude hypoxia (8 h/day; 28 days) showed that vitamin B6, B12, folate, and choline O_2_ can mitigate the deleterious effects of hypoxia exposure on memory [129].

### 4.4. Obstructive Sleep Apnea

To improve our understanding of chronic intermittent normobaric hypoxia, we have included another type of hypoxia, termed OSA, which is the most prevalent sleep disorder and is characterized by the cessation of the continuous flow of air in the upper airways, resulting in repetitive short cycles of hypoxia/normoxia during sleep [134,135]. Several investigations have linked OSA to neurodegenerative diseases, specifically PD and AD [136,137,138,139]. OSA is a comorbidity in more than half of AD patients, and coincidentally, AD patients and individuals with OSA exhibit substantial accumulation of tau proteins [18,19,132].

In addition, OSA triggers molecular pathways related to tau phosphorylation and specific markers of neurodegenerative diseases and aging in in vitro and in vivo models. The levels of biomarkers such as tTau, pTau (at Ser 199/202/214/396/404 and Thr 205/212/231), and IL-6, the total content of Aβ and Aβ42, and the Aβ40/42 ratio have been shown to significantly increase in murine and cell models of OSA and OSA patients [101,140,141,142,143]. In addition, OSA accelerates amyloid and tau pathology through the hyperphosphorylation of tau [77] and affects the activity of p35, a CDK5 activator [76]. Recent studies have shown a close relationship between cognitive function and oxidative stress, which can occur in OSA [144,145].

Considering all the information presented, it is necessary to further study different models of hypoxia because it seems that hypoxia can be damaging or beneficial depending on certain factors, such as the duration of exposure and the O_2_ level. These findings suggest that whether hypoxia exerts a neurodegenerative or neuroprotective effect is dose- and time-dependent; therefore, additional studies on the effects of hypoxia are necessary.

## 5. Biomarkers of Tauopathies

In tauopathies, there is a period prior to official clinical diagnosis called the early or prodromal phase, during which the disease emerges and associated biochemical changes occur. In AD, PD, and HD, this stage can begin 20 years before symptoms appear and patients meet the criteria for disease diagnosis [6,146].

A clinical diagnosis is made after conducting neuropathological studies and psychometric tests combined with imaging [147], which is used to confirm or rule out a specific disease. After diagnostic confirmation, the patient receives treatment aimed at alleviating symptoms, such as drugs or psychological therapy. However, to our knowledge, no treatment has been found to effectively mitigate any of the aforementioned tauopathies; thus, the treatment of these diseases represents a great challenge [148].

In tauopathies, the concentrations of tau and other peptides are altered, directly causing neuronal damage, amyloid deposition, inflammation, brain atrophy, increased permeability of the blood–brain barrier, and the progressive cognitive deterioration characteristics of dementia [12,93,149,150,151,152]. The patterns of NFT formation and Aβ aggregation differ throughout the disease; unlike Aβ aggregates, tau aggregates form in various brain structures in a regular hierarchical pattern, with the affected brain region showing a strong correlation with the disease stage [153]. Therefore, tau can be considered the main indicator of disease pathology during the prodromal phase [153,154].

Diagnostic studies have been performed to measure the levels of certain biomarkers related to neuronal damage, and great efforts have been made to validate their usefulness as biomarkers and incorporate them in routine neurological examinations [155]. These biomarkers are categorized as basic and specific biomarkers: basic biomarkers reflect inflammation, and specific biomarkers are used to directly monitor neuropathological development [152].

As discussed previously in this paper, hypoxia is one of the main stressors that contributes to the development or progression of tauopathies, which is why research has been carried out on the link between hypoxia and tauopathies. We explored the effect of hypoxia on the levels of protein biomarkers of tauopathies as well as markers of neuronal, axonal, and glial damage and inflammation, which typically occur in these diseases (Table 2).

## 6. Controversies

pTau levels reflect the formation of NFTs, and in the context of hypoxia, they reflect neuronal damage caused by O_2_ deprivation. However, there are some inconsistencies regarding changes in pTau levels in patients with Creutzfeld–Jakob disease and acute cerebrovascular accidents, in which high levels of tTau but not pTau are found in CSF [150]; this finding is inconsistent with those of other studies (included in this paper), which have consistently found that O_2_ deprivation increases the phosphorylation of tau. Notably, in several tauopathies, an increase in pTau levels has not been detected, and a moderate increase in tau levels is characteristic of the most common tauopathies, such as AD [44].

These findings suggest that some tauopathies are not directly related to tau hyperphosphorylation and are probably not related to hypoxia; this limits the generalizability of our findings. To our knowledge, there is not much information regarding tau and its behavior in different types of hypoxia and tauopathies, especially in hypobaric hypoxia, except in relation to the link between OSA and AD; therefore, further investigation is needed. It is likely that several of these diseases involve a different mechanism, and although phosphorylation is the main posttranslational modification of tau, it is not the only alteration that contributes to tauopathies.

## 7. Conclusions

It has been found that hypoxic stimuli play important roles in the development of neurodegenerative diseases through different mechanisms, causing a series of phenomena that contribute to neurodegeneration as well as common diseases and medical conditions.

Hypoxia exposure contributes to tau hyperphosphorylation, which is related to the development of NFTs in neuronal cells, leading to neurodegeneration; this neurodegeneration leads to the development of tauopathies, in which the hippocampus seems to be the starting point of disease pathology. In particular, hypobaric hypoxia exposure increases the activation of several kinases related to the hyperphosphorylation of tau, such as ERK, GSK3β, and CDK5. In addition, hypoxia increases the levels of inflammatory molecules (IL-β1, IL-6, and TNF-α), which are also associated with neurodegeneration. Therefore, exposure to high altitudes could contribute to the development of tauopathies.

Notably, to our knowledge, research on hypobaric hypoxia and tauopathies is very scarce, and most related studies have been conducted outside South America; therefore, there is a gap in knowledge that needs to be explored in greater detail. Importantly, at present, the exact etiology of tauopathies and the contribution of hypobaric hypoxia to tauopathy development at the molecular level have not been fully clarified, and additional research is still needed on this topic. This research provides a reference for future studies on this specific topic, which may contribute to the early detection and effective treatment of these diseases.

## Figures and Tables

**Table 1:** Main characteristics of various tauopathies.

Classification	Characteristics	References
Primary tauopathy		
Pick’s disease (PD) frontotemporal dementia	Behavioral changes, social disinhibition, eating disorder, absent/late parkinsonism.	Meeter et al. [35]
Argyrophilic grain disease (AGD)	Personality changes, emotional imbalance, memory impairment.	Rodriguez and Grinberg [36]
Corticobasal degeneration (CBD)	Impairment of cognitive and sensory function, motor and visual disturbances, behavioral changes, aphasia, dysphagia.	Koga et al. [37] and Valentino et al. [38]
Progressive supranuclear palsy (PSP)	Apathy, anxiety, sleep disturbance, spectrum from pure motor presentation to pure cognitive presentation.	Jackson et al. [24]
Creutzfeld–Jakob disease (CJD)	Severe mental decline and motor disturbances.	Vacca [39]
Secondary tauopathy		
Alzheimer’s disease (AD)	Cognitive impairment, psychiatric symptoms, depression.	Plowey and Ziskin [40]
Parkinson’s disease (PD)	Chorea, dystonia, motor impairment, cognitive decline, depression.	Jin et al. [41]
Huntington’s disease (HD)	Motor, cognitive, and psychiatric impairment, OCD, psychosis, depression.	Rawlins et al. [42]
Down’s syndrome	Hypotonia, atlantoaxial instability, reduced neuronal density, cerebellar hypoplasia, intellectual disability, congenital heart defects.	Antonarakis et al. [43] and Arendt et al. [3]

**Table 2:** Protein biomarkers of tauopathies and the techniques used to detect them.

Biomarker	Origin	Technique	Tracer	Disease	Results	References
Tau						
tTau	Plasma	Simoa Quanterix^®^	-	AD	Transient hypoxia increases tTau levels acutely, indicating the potential involvement of tTau in AD.	Gren et al. [122]
tTau	CSF	Elecsys^®^ Electrochemiluminescence	-	AD	tTau levels increase as soon as amyloid pathology appears.	Mila-Alomá et al. [154]
pTau (T181)			-			
pTau (T181)	CSF	ELISA	-	DAT	Altered levels of pTau (T181) are related to dementia progression.	Snider et al. [156]
tTau and pTau (T181)	Plasma	ELISA	-	OSA/AD	tTau and pTau (T181) are found at significantly high levels in OSA patients and are a risk factor for AD in OSA patients.	Kong et al. [143]
pTau (T181)	Plasma	Simoa Quanterix^®^	-	AD	pTau T181 is effective in differentiating between different early symptomatic stages of Alzheimer’s-type dementias.	Gerards et al. [157]
pTau	Histological brain slice	HISTELIDE (immunohistochemistry)	AT8	AD	pTau images with flortaucipir show changes consistent with those observed by histopathological analyses, reflecting the distribution of tau throughout the neocortex. Therefore, flortaucipir is effective in detecting neurofibrillary degeneration caused by pTau.	Pontecorvo et al. [158]
tTau and pTau (T181)	CSF	INNO-BIA AlzBio3, INNOTEST, and xMAP Luminex Fluorimetric Immunoassay	-	AD	The NfL level in CSF and plasma has almost the same diagnostic capacity as pTau and tTau levels in CSF.	Chen et al. [159]
pTau (T181)	Plasma	Simoa Quanterix^®^	-	AD	pTau in plasma is a specific, scalable, and accessible marker of AD progression superior to NfL.	Moscoso et al. [160]
Tau	Plasma	Simoa Quanterix^®^	-	AD		Zhang et al. [161]
pTau (T217)	CSF	PET	[F18] Flortaucipir	AD	pTau (T217) is better at distinguishing between individuals with AD and those with other forms of dementia than pTau (T181).	Janelidze et al. [162]
pTau (T181)	-	PET	[F18] Flortaucipir	HD	The accumulation of pTau (T181) is one of the characteristics of HD.	Giehl et al. [163]
pTau (T181)	-	PET	[F18] Flortaucipir	AD	pTau images with flortaucipir show changes consistent with those observed by histopathological analyses, reflecting the distribution of tau throughout the neocortex. Therefore, flortaucipir is effective in detecting neurofibrillary degeneration caused by pTau.	Pontecorvo et al. [158]
Aβ						
Aβ42	Plasma	Simoa Quanterix^®^	-	AD	Transient hypoxia increases tTau levels acutely, indicating the potential involvement of tTau in AD.	Gren et al. [122]
Aβ42	Plasma	ELISA	-	DAT	Altered levels of Aβ42 are related to dementia progression.	Snider et al. [156]
Aβ40 and Aβ42	Plasma	ELISA	-	AD	Aβ40 and Aβ42 are found at significantly high levels in OSA patients.	Kong et al. [143]
Aβ42	CSF	Electrochemiluminescence NeuroToolKit^®^	-	AD	Aβ42 is the first marker whose level is altered in the preclinical phase of AD.	Mila-Alomá et al. [154]
Aβ42/Aβ40 ratio	plasma	Simoa Quanterix^®^	-	AD	The Aβ42/Aβ40 ratio can robustly differentiate groups of patients with preclinical AD.	Gerards et al. [157]
Aβ42	Histological brain slice	HISTELIDE (immunohistochemistry)	H31L21	AD	pTau images with flortaucipir show changes consistent with those observed by histopathological analyses, reflecting the distribution of tau throughout the neocortex. Therefore, flortaucipir is effective in detecting neurofibrillary degeneration caused by pTau.	Pontecorvo et al. [158]
Aβ	-	PET	[F18] Florbetapir	AD	Elevated Aβ levels in clinically normal elderly people are associated with poorer cognitive performance, similar to those observed in the early stages of the disease.	Sperling et al. [164]
Aβ	CSF	PET	[F18] Flutemetamol	AD	pTau (T217) is more strongly related to amyloid pathology than is pTau (T181).	Janelidze et al. [162]
Other						
S100-β	Plasma		-	AD	There is no increase in S100-β levels under hypoxia.	Gren et al. [122]
NfL	Plasma		-	AD	There is no increase in NfL levels under hypoxia.	Gren et al. [122]
NfL	CSF	ELISA	-	HD	NfL is useful as a biomarker for HD and can be used to differentiate patients in different disease stages.	Niemelä et al. [165]
NfL	CSF	Electrochemiluminescence NeuroToolKit^®^	-	AD	NfL levels increase with age, regardless of the presence of amyloidosis.	Mila-Alomá et al. [154]
Ng			-			
NfL	Plasma	Simoa Quanterix^®^	-	AD	NfL levels have a strong correlation with age.	Gerards et al. [157]
NfL	CSF	NF-light^®^ ELISA	-	AD	NfL levels are significantly higher in individuals with AD, and NfL levels in CSF and plasma have almost the same diagnostic ability as pTau and tTau levels in CSF.	Chen et al. [159]
	Plasma	Simoa Quanterix^®^	-			
NfL	Plasma	Simoa Quanterix^®^		AD	NfL is a nonspecific marker of AD that can be used as a marker of neuronal deterioration.	Moscoso et al. [160]
Ng	CSF	ELISA, HI-MS	-	AD	Ng can be considered a routine biomarker for AD prognosis.	Kvatsberg et al. [166]
NfL	Plasma	Simoa Quanterix^®^	-	AD	NfL levels are positively related to AD.	Zhang et al. [161]

pTau: hyperphosphorylated tau protein; tTau: total tau protein; Aβ: β-amyloid protein; Aβ40: β-amyloid 40 protein isoform; Aβ42: β-amyloid 42 protein isoform; Aβ42/40: amyloid protein ratio 42/40; NfL: neurofilament light chain protein; Ng: neurogranin; CSF: cerebrospinal fluid; ELISA: enzyme-linked immunosorbent assay; PET: positron-emission tomography; HI-MS: mass spectrometry.

## Data Availability

Not applicable.
